# Vesicles: a supramolecular tool to achieve organic reactions in aqueous media

**DOI:** 10.3389/fchem.2026.1761290

**Published:** 2026-03-06

**Authors:** Anshupriya Shome

**Affiliations:** Department of Chemistry, Brahmananda Keshab Chandra College, Kolkata, India

**Keywords:** aqueous core, bilayer membrane, enzymes, green chemistry, organic reactions in water, supramolecular aggregates, vesicle

## Abstract

Vesicles are fascinating supramolecular aggregates that can provide an interface-rich microenvironment and confined space. They can incorporate hydrophilic materials in their aqueous core and hydrophobic materials in their bilayer membrane. Thus, they can be utilized to perform organic reactions in water. Using water as a solvent for organic reactions instead of hazardous organic solvents is one of the important strategies of green chemistry. Several factors, including aggregate structure and composition, nature of the substrate, and reaction conditions, determine whether an organic reaction will be catalyzed or inhibited. Enzyme-containing vesicles can also be used as nanoreactors for organic reactions in water. Reactions in vesicles have been extensively studied over the past few decades, and their applications have been explored. A better understanding of the effect of vesicles on organic reactions will make the design of green organic reactions easier. In this study, related research works have been discussed.

## 1 Introduction

Supramolecular aggregates always produce organization from chaos, as nature does. In supramolecular systems, individual molecules self-assemble to form higher-order aggregated structures with amazing features. One of the astonishing supramolecular assemblies created by nature is the cell membrane. Self-aggregation of lipids creates compartmentalization of cellular organisms within cells, making otherwise incompatible reactions possible. Of the different types of supramolecular assemblies, the vesicle is the one that has the closest resemblance to the cell membrane. For decades, they have been utilized for encapsulation and cellular delivery of probes, drugs, and DNA, as well as models for cell membranes (Alenzi et al., 2023; De Jong et al., 2019; Zhao et al., 2024). Apart from these conventional vesicle applications, they can also be used to achieve organic reactions in water. The use of water as a solvent for organic reactions is simultaneously advantageous and challenging. Water is a perfect solvent for green chemistry, as it is non-toxic, inexpensive, and environmentally friendly (Breslow, 2010; Hartonen and Riekkola, 2017; Jung and Marcus, 2007; Sheldon, 2005). However, a major disadvantage of using water as a solvent for organic reactions is that most organic substrates are insoluble in water. To overcome this problem, dispersed systems like polyelectrolyte solutions, micelles, and vesicles comprised of surfactants have been used as solvents for organic reactions (Kuchler et al., 2016; Peacock and Blum, 2023; Ruiz-Lopez et al., 2020; Serrano-Luginbühl et al., 2018). These systems consist of an interface that is markedly different from both the core structure and the aqueous medium in which they are dissolved or dispersed (Serrano-Luginbühl et al., 2018). These interfaces can accelerate the organic reactions by increasing the solubility of hydrophobic compounds in water (Peacock and Blum, 2023). In this regard, vesicles have gained special attention in both basic and applied sciences due to their resemblance to cell membranes (Serrano-Luginbühl et al., 2018; Walde et al., 2014). In vesicles, the chances of solubilization and compartmentalization of organic substrates are enhanced, making them suitable media for controlling the reaction (Cabaleiro-Lago et al., 2006). In some instances, they can catalyze organic reactions (Klijn and Engberts, 2003; Paprocki et al., 2015; Pérez-Juste et al., 2000; Rispens and Engberts, 2001). Three major effects can contribute to the reaction rate enhancement in vesicle systems. These reasons are increased concentration of the reactants in the vesicular aggregates, difference in polarity at the reaction site, and reduction in probability of side reactions due to enhanced steric hindrance (Paprocki et al., 2015). An efficient catalysis is obtained when oppositely charged hydrophobic substrates bind to charged vesicles and other reactants come in proximity of the substrate as a counterion to the aggregate (Rispens and Engberts, 2001). In contrast, vesicles can also inhibit chemical reactions when only one of the two reactants is encapsulated within the vesicle (Hervés et al., 1997; Moss et al., 1988). In the past few decades, organic reactions in vesicles have been extensively studied to replace the toxic organic solvents with ecofriendly water. In addition, the reactions in vesicular aggregates can mimic the chemical reactions at complicated biological membranes to some extent. While discussing organic reactions in aqueous media, we must not forget the enzymatic bio-catalysis in water. In living systems, many enzymatic reactions happen at cell membranes (Conrado et al., 2008; Kuchler et al., 2016; Satyawali et al., 2017). For example, soluble enzymes acting on membrane-bound substrates are kinases, phosphatases, and GTPases (Leonard et al., 2023). 5′-Nucleotidase catalyzes the hydrolysis of nucleotides at the membrane as well as cytoplasm by releasing phosphate from the 5′-position of the pentose ring (Kato et al., 1979). Sphingomyelinase at the membrane helps in breaking down sphingomyelin into ceramide, which is crucial for cell signaling, membrane function, and lipid metabolism (Goñi and Alonso, 2002). Cell-membrane-expressing enzymes, such as metalloproteinases, hydrolyze at the cell surface, playing crucial roles in cell growth, intercellular communication, cell migration, inflammation, and cancer pathogenesis (Gorry et al., 2020). Such reactions can be experimentally analyzed in the vesicle system (Blocher et al., 1999; Deshwal and Maiti, 2021; Jiang et al., 2025; Kato et al., 2003; Li et al., 2007; Luna et al., 2025; Walde and Ichikawa, 2001). Vesicles can protect enzymes from harmful external conditions (Trenkenschuh et al., 2022; Yoshimoto, 2016). Vesicles containing enzymes can also be utilized to develop cell-like microreactors as artificial cells (Lu et al., 2022; Ridgway-Brown et al., 2025; Seo and Lee, 2022; Shin and Jang, 2024). It is very important to understand the role of vesicular systems in organic reactions under chemo-catalysis and enzyme-catalysis conditions in aqueous solvent. In this regard, a review article that includes the updated research works in this field is indeed required to explore organic reactions in a vesicle system and apply them. In this review, we will discuss different research works with the aim of understanding how the vesicular system can influence different organic reactions in water.

## 2 Vesicle aggregates as a host for organic reactions

**FIGURE 1:**
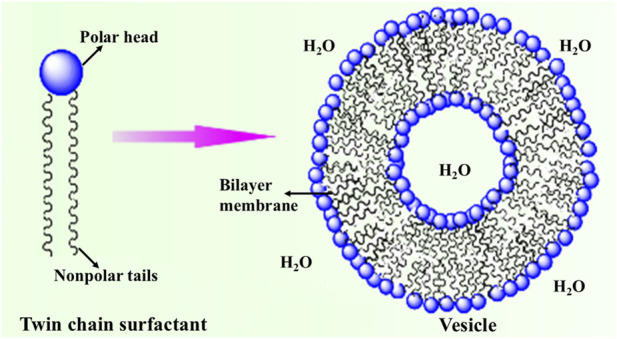
Schematic representation of vesicles.

Vesicles are spherical aggregates where the aqueous compartments are enclosed by a bilayer membrane of twin-chain surfactants with both polar ends facing the aqueous medium (Figure 1).

**FIGURE 2:**
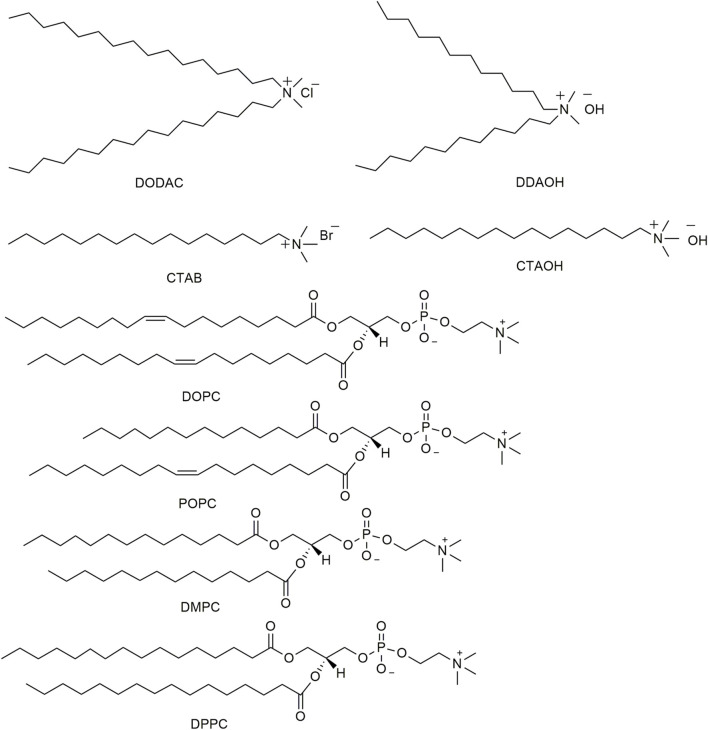
Chemical structure of various surfactants used in the study.

Double-chain surfactants like phospholipids are the basic building blocks of all biological membranes. Other artificial twin chain surfactants can also form synthetic vesicles (Figure 2). Due to the presence of an aqueous core and a lipid bilayer, vesicles can incorporate both hydrophilic and hydrophobic molecules. Whether the vesicle will catalyze or inhibit different organic reactions will be regulated by the aggregate structure and composition, the nature of the substrate, reaction conditions, and the location of reagents in different parts of the aggregate.

### 2.1 Hydrolysis reactions

#### 2.1.1 Ester hydrolysis

The N-nitroso compounds have biological importance because a wide variety of structurally related compounds having the N-nitroso-N-alkyl functional group have shown anti-cancer properties (Castro et al., 1986; Fernández et al., 2015). The mechanism of acid- and base-catalyzed hydrolysis of N-nitroso compounds is well known (Castro et al., 1989).

**FIGURE 3:**
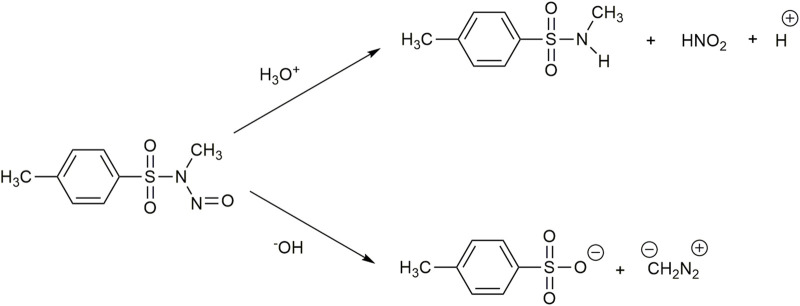
Mechanisms for acid and alkaline hydrolysis of methyl-N-nitroso-p-toluenesulfonamide (MNTS) in water.

In this regard, acid and alkaline hydrolysis of N-methyl-N-nitroso-*p*-toluenesulfonamide (MNTS) (Figure 3) in a vesicle host comprised of dioctadecyldimethylammonium chloride (DODAC) (Figure 2) surfactant was performed by Hervés et al. (1997). The cationic vesicles were prepared by sonication. In the acid-catalyzed hydrolysis process, the slow step is the proton transfer from the solvent to the substrate, whereas in alkaline hydrolysis, it is the nucleophilic attack of the hydroxyl anion on the sulfur atom (Figure 3). The hydrodynamic diameter (Dh) of the DODAC single-compartment vesicles used was found to be 40 nm, and a polydispersity of 0.10 was obtained. According to their explanation, the hydrolysis reaction occurs in both DODAC bilayers of the vesicular pseudo-phase as well as in the aqueous phase comprising the bulk medium and the intravesicular compartment. The overall reaction rate has a contribution from the reaction rate in both pseudo-phases.

MNTS distribution in both phases maintained an equilibrium. The MNTS association constant in the bilayer increased with the increase in the number of carbon atoms in the surfactant, indicating that hydrophobic forces are responsible for the association of MNTS in bilayers. In acid hydrolysis, the rate constant decreased with an increase in the DODAC concentration. The proton concentration at the DODAC bilayer was very low due to electrostatic repulsion, as the vesicle is cationic in nature. It may be that the reaction took place solely in the aqueous pseudophase, reducing the overall rate of acid hydrolysis. In contrast, in basic hydrolysis, the reaction rate increased with an increase in DODAC concentration (Table 1). In this case, the cationic bilayer surface attracted hydroxyl ions at the vesicular pseudo-phase. OH^−^ ions in both sides of the bilayer showed comparable reactivity (Cuccovia et al., 1989). Thus, the overall reaction rate, which was the sum of the rates at the vesicular and aqueous pseudo-phases, increased. The catalytic efficiency of basic hydrolysis of MNTS in the presence of DODAC vesicles was found to be 25 times higher than that in CTAB micellar systems (Hervés et al., 1997).

**TABLE 1:** Kinetic parameters for hydrolysis in water.

Reaction	Reagent condition	Kinetic parameter	Kinetic parameter	Reference
Acid hydrolysis MNTS in water in 4.0 × 10^−3^ M DODAC	2.50 × 10^−3^ [H^+^]/M	kw/M^−1^ s^−1^ = 0.031	*K*_MNTS/M_^−1^ = 370	Hervés et al. (1997)
Base hydrolysis MNTS in water in 4.0 × 10^−3^ M DODAC	2.50 × 10^−3^ [OH^−^]/M	kw/M^−1^ s^−1^ = 0.083	*K*_MNTS/M_^−1^ = 380	
Rate constants for the first phase of the basic hydrolysis of diazepam (1a, Figure 4) in cetyltrimethylammonium hydroxide (CTAOH)	Surfactant concentration = 4 mM NaOHlmM = 0	K_1_/s^−1^ = 1.24 × 10^−3^	​	Broxton et al. (1988)
	Surfactant concentration = 50 mM NaOHlmM = 0	K_1_/s^−1^ = 8.18 × 10^−3^	​	
Rate constants for the first phase of the basic hydrolysis of diazepam (1a, Figure 4) in didodecyldimethylammonium hydroxide (DDAOH)	Surfactant concentration = 4 mM NaOHlmM = 0	K_1_/s^−1^ = 1.16 × 10^−3^	​	
	Surfactant concentration = 50 mM NaOHlmM = 0	K_1_/s^−1^ = 9.64 × 10^−3^	​	

#### 2.1.2 Basic hydrolysis of benzodiazepinones

**FIGURE 4:**
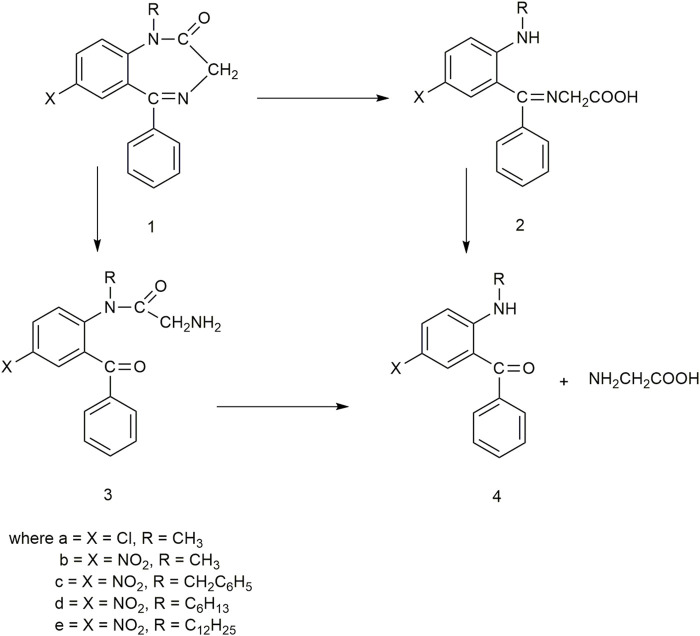
Basic hydrolysis of diazepam (la) and N-alkyl nitrazepam derivatives (1b–e).

Benzodiazepinones are hydrophobic compounds with anxiolytic, anticonvulsant, and muscle-relaxing properties. They have been widely used as psychoactive drugs that act as central nervous system depressants (Oliveira and Chaimovich, 1993). Broxton et al. (1988) observed the hydrolysis of benzodiazepinones in vesicles prepared from didodecyldimethylammonium hydroxide (DDAOH) as well as in micelles of cetyltrimethylammonium hydroxide (CTAOH) (Figure 2). The hydroxide counterion of the surfactants can contribute to the basic hydrolysis of the substrate. The hydrolysis of nitrazepam, a 1,4-benzodiazepinone derivative, involves initial amide hydrolysis (Figure 4, 1a to e).

An increase in absorbance at 252 nm and 385–390 nm indicated the formation of *p*-chloroaniline chromophore 2a from compound la and a *p*-nitroaniline chromophore 2b–e from all the other compounds. Rate constants for the hydrolysis of diazepam la in micelles of CTAOH and in vesicles of DDAOH were found to be increased with surfactant concentration. When the CTAOH concentration was 4 mM, the rate constant of reaction was 1.24 × 10^−3^s^−1^. With increasing concentration of CTAOH, the rate constant also increased up to 8.18 × 10^−3^s^−1^ at 50 mM. At 4 mM DDAOH, the rate constant of reaction was 1.16 × 10^−3^s^−1^, and it increased to 9.64 × 10^−3^s^−1^ at 50 mM (Table 1). This enhancement may be attributed to either an increase in the hydroxide ion concentration or an increase in the proportion of reaction in the surfactant aggregate. However, for all substrates, the rate of hydrolysis increased when the surfactant concentration was increased at a constant total hydroxide concentration (0.1 M) and when the concentration of added NaOH was increased at constant surfactant concentration (12 mM). They found that the dependence of DDAOH concentration in the range 1–5 mM varies from a 2.5-fold increase for compound le to a 6-fold increase for compound 1b. The compounds containing hydrophobic alkyl groups (1d and e) showed the smallest increase in rate with an increased concentration of DDAOH. It is possible that hydrophobic alkyl chains at N-1 in compounds 1d and le resulted in the penetration of the amide group more deeply into the hydrophobic region of the aggregate, making it less sensitive to changes in the bulk solution. In contrast, compound lb, with only the methyl group at N-1, experiences a larger increase in rate. The phenyl group on C-5 resulted in residing the amide moiety at the interface, making it more sensitive to changes in bulk solution.

### 2.2 S_N_2 reaction

**FIGURE 5:**
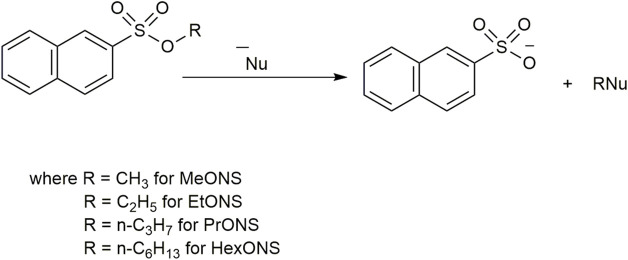
S_N_2 reaction of 2-alkylnaphthtalenesulfonates (AlkONS) with nucleophiles.

Studies about the solvent effects on S_N_2 reactions began many years ago, but it is still a topic of recent interest (Ingold, 1969; Pliego, 2021). In this regard, Klijn and Engberts (2005) studied the vesicular catalysis of S_N_2 reactions for a series of aromatic alkyl sulfonates (Figure 5) with water and bromide as nucleophiles.

These substrates were chosen due to their hydrophobicity, which makes them reactive in the presence of micelles and vesicles. As a host, they have used different types of vesicles prepared from di-*n*-hexadecyldimethylammonium bromide (C_16_C_16_+) and mixed vesicles of C_16_C_16_+ and 50 mol% of *n*-dodecyl-*s* glucoside (C_12_Glu) and of 1-palmitoyl-2-oleoyl-*sn*-glycero-3-phosphocholine (POPC). The preparations were referred to as 0:100:0 and 0:50:50 compositions, where the numbers denote the mole fractions of POPC, C_16_C_16_+, and C_12_Glu, respectively. However, 0:100:0 vesicles were unstable and precipitated after an hour of preparation. To overcome this problem, the authors prepared more stable mixed vesicles with POPC, where POPC can control the counterion binding by external addition of NaBr as well as maintain the constant surface charge density by changing the composition. The observed rate of the reaction was considered as the sum of the rates in the aqueous and vesicular pseudo-phases for both the reactions with water and bromide ions. Therefore, a total of four rate constants must be considered. Two of them (k′_H2O,w_ and k′_Br_^−^_,w_) are pseudo-first-order rate constants of the reaction with, respectively, water and bromide ions in the aqueous pseudo-phase. The other two (k′_H2O,ves_ and k′_Br_^−^_,ves_) are the pseudo-first-order rate constants of the reaction with, respectively, water and bromide ions in the vesicular pseudo-phase. The C_12_Glu was found to be a poor nucleophile, so its reaction with the substrate was not considered (Galema et al., 1992). When 2-alkylnaphthtalenesulfonates (AlkONS) are used as a substrate in the presence of 80:20:0 vesicles, k_obs_ increased. Although the concentration of water in the Stern region was lower than that in the bulk, water molecules in the Stern region are more reactive toward MeONS (Figure 5). Isotopic labeling suggested that there was no change in the reaction mechanism on going from water to the vesicular surface. When bromide ions are added to the vesicular solution, the reaction with bromide ions is inhibited by a factor of ca. 3 in the vesicular pseudophase, irrespective of the presence of C_12_Glu. This observation proved that the reaction with bromide ions is less favorable in the presence of vesicles than that in water (Table 2). A similar trend was also observed in the presence of 35:15:50 vesicles but to a lesser extent.

**TABLE 2:** Kinetic parameter of S_N_2 reaction of MeONS with water and bromide ions in a mixed vesicular system comprising of di-*n*-hexadecyldimethylammonium bromide (C_16_C_16+)_, *n*-dodecyl-*s* glucoside (C_12_Glu), and 1-palmitoyl-2-oleoyl-*sn*-glycero-3-phosphocholine (POPC).

vesicle	[NaBr] (mM)	*k*H_2_O,ves (M^−1^ s^−1^)	*k*Br^−^,ves (M^−1^ s^−1^)	*K*_S_ (M^−1^)	*k*H_2_O,ves /*k*H_2_O,w	*k*Br^−^,ves /*k*Br^−^,w
80:20:0	0	8.4 × 10^−6^	​	*656*	10.3	-
	100	8.4 × 10^−6^	7.4 × 10^−5^	704	10.3	0.31
	200	8.4 × 10^−6^	1.8 × 10^−4^	628	10.3	0.74
35:15:50	0	4.8 × 10^−6^	​	*453*	6.0	-
	100	4.8 × 10^−6^	6.8 × 10^−5^	436	6.0	0.28
	200	4.8 × 10^−6^	8.8 × 10^−5^	470	6.0	0.38
100:0:0	0	5.3 × 10^−7^	​	349	0.66	-
	100	5.3 × 10^−7^	7.4 × 10^−5^	349	0.66	0.31
	200	5.3 × 10^−7^	1.8 × 10^−4^	349	0.66	0.74
50:0:50	0	3.7 × 10^−7^	​	251	0.38	-
	100	3.7 × 10^−7^	6.8 × 10^−5^	251	0.38	0.28
	200	3.7 × 10^−7^	8.8 × 10^−5^	251	0.38	0.38
80:20:0	100	3.9 × 10^−7^	3.9 × 10^−5^	800	0.61	0.27

No catalysis was observed in vesicles without C_16_C_16_+, or in the 100:0:0 and 50:0:50 vesicles. These observations indicated that the presence of cationic amphiphiles is required for an increase in the reactivity of water. While comparing the reactions in zwitterionic vesicles with a composition 100:0:0 and 50: 0:50, inhibition of reaction was observed. This may be due to the dilution of charges because counterion binding is more efficient for vesicles where the zwitterionic headgroups are not diluted by the presence of C_12_Glu. With an increase in substrate chain length, the observed reaction rate constants were found to be different in the micellar and vesicular hosts.

### 2.3 Oxidation

#### 2.3.1 Oxidation of Ellman’s anion

**FIGURE 6:**
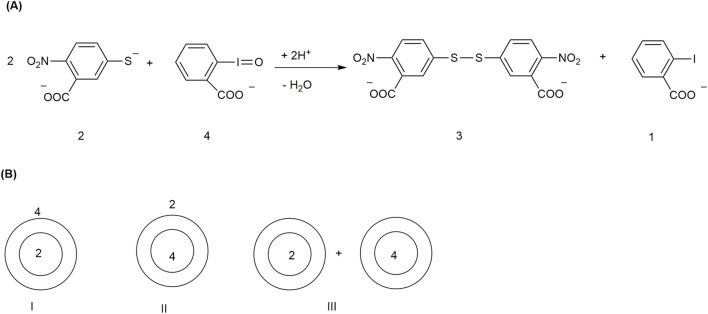
**(A)** Conversion of Ellman’s anion (2) to Ellman’s reagent (3) by *o*-iodosobenzoate (4). **(B)** Reaction configurations for the oxidative dimerization of Ellman’s anion (2) by *o*-iodosobenzoate (4) in the presence of DODAC vesicles.

Ellman’s reagent, or 5,5′-dithiobis-(2-nitrobenzoic acid) (DTNB), is a water-soluble reagent that reacts with thiols rapidly and sensitively. Therefore, it can be used to determine the thiol content of proteins and biological tissues (Riddles et al., 1983; Ginet et al., 2025). Moss et al. (1988) used vesicles of dioctadecyldimethylammonium chloride (DODAC (Figure 2) as a host to control oxidative dimerization reaction of Ellman’s anion (2) by *o*-iodosobenzoate (4). The oxidation product is Ellman’s reagent (3) (Figure 6A).

The product formation was monitored by decay of the UV absorption of 2 (ʌ_max_ 412 nm, € = 13,600 M^−1^ cm^−1^) and increase in the absorption of its disulfide oxidative dimer, Ellman’s reagent (3) at 332 nm (€ = 19,860 M^−1^ cm^−1^). When Ellman’s anion (2) and *o*-iodosobenzoate (4) are in the same aqueous solution, they react within 10 s, but if any one of the reagents is encapsulated in a vesicle, the reaction rate decreases. In any one of the situations described in Figure 6B, the reaction rate is decreased. Encapsulated reagent is unavailable to an anionic substrate or *vice versa*. The most important observation was that when the reagents were entrapped separately in DODAC vesicles and taken in the same aqueous solution for many hours, the reaction rate was very slow, which can be effectively applied in the storage of reagents in the same solution without reaction. The reaction could be achieved at the required time by the addition of a reagent like ethanol or by warming the solution to 38^ °^C, which destroyed the vesicle and allowed the reagents to contact each other. In another work, a platinum-supported catalyst combined with phosphatidylcholine vesicles was used to convert glycerol to lactate under mild alkaline conditions and at 60 °C. The yield was found to be 90% within 24 h (Table.3) (Shimanouchi et al., 2023).

**TABLE 3:** Yield of the oxidation and multicomponent reactions in different solvents.

Name of the reaction	Solvent system	Yield	Reference
Oxidation of glycerol to lactic acid using a platinum-supported catalyst	Catalyst combined with phosphatidylcholine vesicles	90% within 24 h	Shimanouchi et al. (2023)
Photooxidation of α-pinene (α-PE) sensitized by 9,10-dicyanoanthracene (DCA)	Catanionic vesicles containing an equimolar mixture of *n*-octyltrimethylammonium bromide and sodium laurate	The material balance >95%	Li et al. (2000a)
Light-induced oxidation of reduced nicotinamide adenine dinucleotide (NADH) to NAD^+^ using photosensitizer RuC0 or RuC9	Liposomes consisting of a 100:1 mixture of DPPC and the stabilizing lipid (14:0 PEG 2000 PE)	Reaction is nearly complete within 60 min	Nau et al. (2022)
Oxidation of 4-methoxyphenylboronic acid	Water	60%, even though the reaction time was extended to 96 h	Que et al. (2020)
Oxidation of 4-methoxyphenylboronic acid, 4-propylphenylboronic acid, and 4-hexylphenylboronic acid	Vesicles of PEG-*b*-PPFMA/Ru-Cat (fluorinated)	90%–93% in 20–25 h	
	Vesicles of PEG-*b*-PBMA/Ru-Cat	33%–52% after 30 h	
Multicomponent reaction (MCR): Passerini reaction	Dichloromethane	59% in 24 h	Paprocki et al. (2015)
	PBS (pH 7.4)	33% in 24 h	
	Triton X-100 micelles	47% in 24 h	​
	Dimethyldioctadecylammonium bromide (DDAB) vesicle	48% in 24 h	​
	Dioctadecyldimethylammonium bromide (DODAB) vesicle	58% in 24 h	
MCR: Ugi reaction	Methanol	55% in 48 h	Madej et al. (2017)
	PBS (pH 7.4)	19% in 48 h	
	Triton X-100 micelles	60% in 48 h	
	DDAB vesicle	62% in 48 h	
	DODAB vesicle	42% in 48 h	

#### 2.3.2 Photosensitized oxidations

Selective dye-sensitized photooxidation of alkenes by molecular oxygen is very important, as these reactions are used to produce organic building blocks and intermediates (Hoffmann, 2008; Prein and Adam, 1996). Photosensitization can be applied across a wide range of fields. It is a three-component reaction where all components, light, the photosensitizer (PS), and oxygen, must be present at the same time to induce the related photodynamic effect. By irradiating the sensitizer, reactive oxygen species (ROS) are formed. PSs preferentially accumulate in lipid membranes (Zolcsák et al., 2025). There are two different mechanisms of dye-sensitized photooxidation: the energy-transfer pathway and the electron-transfer pathway (Foote, 1991). In most instances, these two mechanisms of photooxidation are followed simultaneously, resulting in poor selectivity. Organized and constrained hosts for reaction can be used to achieve selectivity (Ramamurthy and Turro, 1995).

**FIGURE 7:**
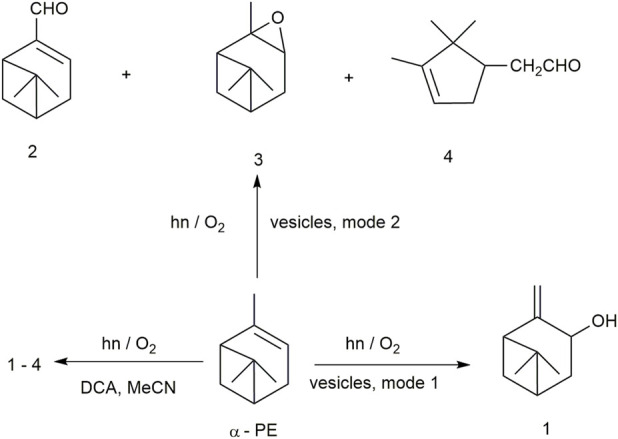
Photooxidation of α-PE sensitized by DCA.

In this regard, Li et al. (2000a) (*Chemical Communications*, 2000) reported photooxidation of α-pinene (α-PE) (Figure 7) and trans,trans-1,4-diphenyl-1,3-butadiene (DPB) sensitized by 9,10-dicyanoanthracene (DCA) in catanionic vesicles containing an equimolar mixture of *n*-octyltrimethylammonium bromide and sodium laurate. The oxidation was done following two different methods. In method 1, the sensitizer DCA was incorporated in the vesicle bilayer, and the substrate was solubilized in a different set of vesicles. Equal volumes of the two sets of vesicle dispersions were then mixed to achieve photolysis. In the second method, both the sensitizer and the substrate were incorporated in the same set of vesicles. The concentrations of the olefins and the sensitizer were 1 × 10^−3^ M and 1 × 10^−4^ M, respectively. The samples were irradiated in the presence of oxygen by light with wavelength λ > 400 nm. The products were extracted with dichloromethane and monitored by gas chromatography. The material balance obtained was >95% (Table 3). Photooxidation of α-pinene sensitized by DCA in homogeneous solution and then reduction of the reaction mixture with sodium sulfite solution gave the ene product pinocarveol 1, and the non-ene products myrtenal 2, epoxide 3, and aldehyde 4 (Figure 7). The ene and the non-ene products are obtained from the energy- and electron-transfer mechanisms, respectively (Tung and Guan, 1998).

The product distribution of the photosensitized oxidation of α-pinene obtained in solvent acetonitrile, dichloromethane, and vesicle systems (Table 4) confirmed the regulating role of the experimental method. Photooxidation in method 1 produced only the ene product 1. Photooxidation in method 2 only gave the non-ene products 2–4. The DCA sensitizer can act through an energy transfer as well as an electron-transfer mechanism (Kanner and Foote, 1992). During the photooxidation of α-pinene in method 1, the α-pinene was isolated from DCA because they were in different sets of vesicles, preventing the electron transfer. However, singlet oxygen can be generated in the DCA-containing vesicles by energy transfer from the triplet excited state of DCA to the ground state of oxygen. ^1^O_2_ with a relatively long lifetime can diffuse freely from one set of vesicles to another, where a reaction occurs with the olefins, producing the ene product 1. In method 2, the DCA molecule was surrounded by many α-pinene molecules in the same set of vesicles, leading to efficient quenching of the singlet excited state of DCA through an electron-transfer process. In this process, DCA radical anions and α-pinene radical cations were generated, and no singlet oxygen-mediated product was produced. The DCA radical anions transferred electrons to oxygen to produce superoxide radical anions, which reacted with α-pinene radical cations located in the same vesicle to yield the products 2–4. Thus, by using confined vesicle systems, we can control the interaction between the substrate and sensitizer molecules in the reaction media and obtain the directed selectivity toward the products. Similar selectivity was also obtained when photosensitized oxidation was performed with DPB as a substrate (Li et al., 2000b).

**TABLE 4:** Product distribution in the DCA-sensitized photooxidation of α-PE.

Media	1	2	3	4
CH_3_CN	52	32	13	3
CH_2_Cl_2_	85	10	5	0
Vesicles (Mode 1)	100	0	0	0
Vesicles (Mode 2)	0	55	4	41

#### 2.3.3 Oxidation of nicotinamide adenine dinucleotide (NADH)

**FIGURE 8:**
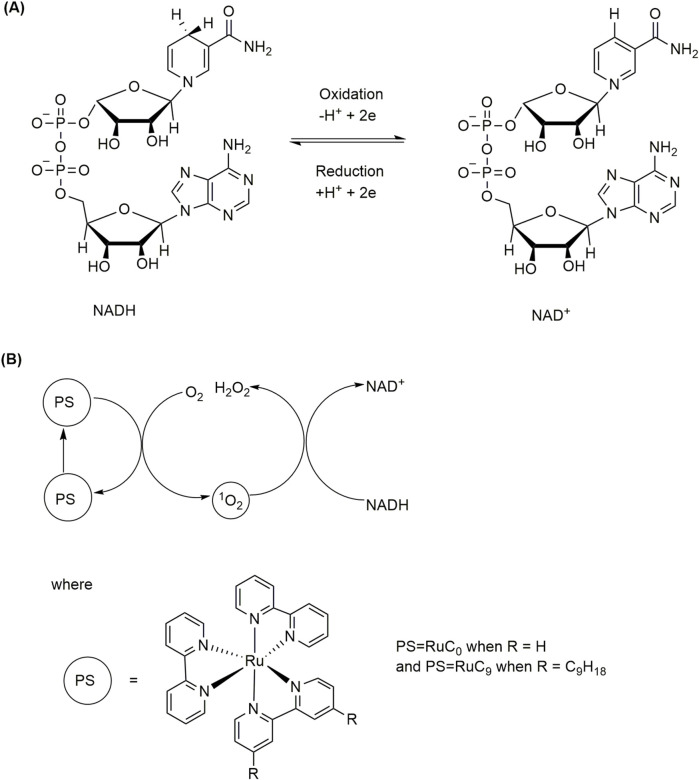
**(A)** Interconversion of NADH and NAD^+^. **(B)** Photosensitized NADH to NAD^+^ conversion.

Nau et al. (2022) showed that encapsulation of photosensitizer (PS) and substrate within liposomes enhanced the photosensitized model reaction of nicotinamide adenine dinucleotide (NADH) conversion to its oxidized form (NAD^+^) compared to classical homogeneous reaction conditions (Figure 8).

Nau et al. (2022) used liposomes consisting of a 100:1 mixture of 1,2-dipalmitoyl-sn-glycero-3-phosphocholine (DPPC) (Figure 2) and the stabilizing lipid 1,2-dimyristoyl-sn-glycero-3-phosphoethanolamine-N-[methoxy(polyethylene glycol)-2000] ammonium salt (14:0 PEG 2000 PE) with 10 mM phosphate buffer. The photosensitizers used were tris(2,2′- bipyridine)-ruthenium(II) chloride (RuC_0_) or bis(2,2′-bipyridine)-(4,4′-dinonyl-2,2′-bipyridine)-ruthenium(II) hexafluorophosphate (RuC_9_) (Figure 8B). The reaction was found to be almost completed within 60 min (Table 3). In addition, the reaction was found to occur approximately 40% faster when the photosensitizer was dissolved in the inner aqueous compartment instead of being embedded within the phospholipid bilayer due to the diffusion behavior of singlet oxygen, which is the oxidant in this reaction. Sinambela et al. (2025) also reported light-induced electron transfer across a lipid bilayer membrane of liposome vesicles through a rigid oligoaromatic molecular wire, which can electronically connect an oxidation and reduction reaction separated by the membrane (Sinambela et al., 2025). The molecular wire can be easily synthesized using benzothiadiazole and fluorene units. It absorbs in the visible spectrum, which makes it suitable for solar energy conversion. Sinambela et al. (2025) studied light-driven NADH oxidation on one side of the membrane and light-driven reduction of an organic water-soluble dye in the bulk phase of liposomes. Most importantly, the system is active in both aerobic and anaerobic atmospheres, so it is ideal for aerobic reactions that produce oxygen, such as solar-driven water splitting and artificial photosynthesis applications. In a recent work, L-α-phosphatidylcholine vesicles with viral receptors encapsulating porphyrin photosensitizers have been applied in antiviral therapies (Horníková et al., 2024).

#### 2.3.4 Photo-mediated aerobic oxidation in fluorinated vesicles

**FIGURE 9:**
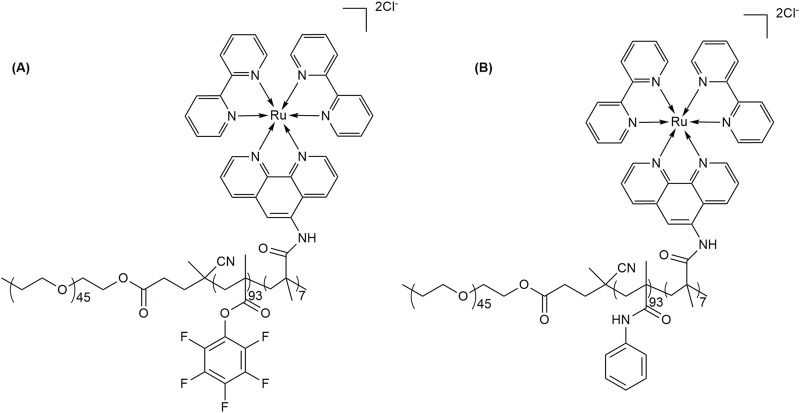
**(A)** Structure of PEG-*b*-PPFMA/Ru(bpy)_2_(phen-NH_2_)Cl_2_; (PPFMA = poly(pentafluorophenyl methacrylate); bpy = bipyridyl and phen-NH_2_ = 5-amine-1,10-phenanthroline) and **(B)** PEG-*b*-PBMA/Ru-Cat (PBMA = poly(benzyl methacrylamide)).

Molecular oxygen is an ideal oxidizing agent, as it is natural, easily available, and environmentally friendly. Various metal-catalyzed organic reactions have been used to synthesize valuable compounds using molecular oxygen as an oxidizing agent (Zhang et al., 2011; Zhang and Jiao, 2010). In this context, Ru-based compounds, including tris(2,2-bipyridine) ruthenium (II) and its derivatives, are used as catalysts for several photo-mediated organic reactions (Prier et al., 2013). However, there are some limitations, such as the use of organic solvents, difficult recovery of the catalyst, and a low reaction rate (Que et al., 2016). To overcome these problems, Que et al. (2020) prepared recyclable fluorinated vesicles implanted with Ru-based catalysts that can act as the nanoreactors for the aerobic oxidative hydroxylation of arylboronic acid. Vesicles with fluorinated membrane embedded with Ru-catalysts were prepared by self-assembly of a PEG-*b*-PPFMA/Ru(bpy)_2_(phen-NH_2_)Cl_2_ amphiphilic copolymer (Figure 9A). To determine the effect of a fluorinated domain around Ru-Cat on oxidation, they also prepared vesicles with PEG-*b*-PBMA/Ru(bpy)_2_(phen-NH_2_)Cl_2_ amphiphilic copolymer (PBMA, poly(benzyl methacrylamide)) (Figure 9B).

**FIGURE 10:**
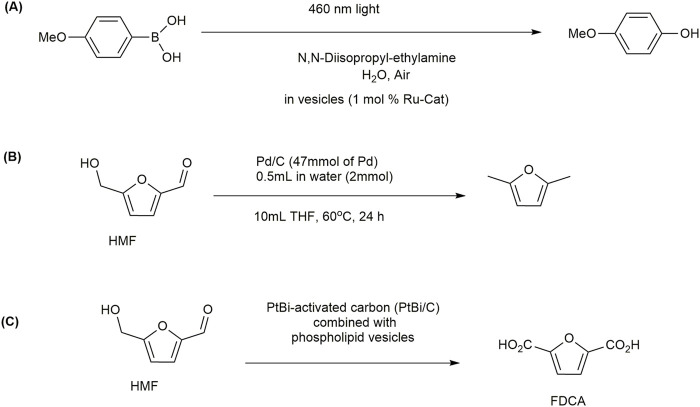
**(A)** Aerobic oxidative hydroxylation of arylboronic acids. **(B)** Conversion of 5-hydroxymethylfurfural (HMF) to 2,5-DMF. **(C)** Conversion of HMF to 2,5-furandicarboxylic acid (FDCA).

To achieve the vesicle, they added 25 mL of H_2_O into dimethylformamide (DMF) solutions of PEG-*b*-PBMA/Ru-Cat and PEG-*b*-PPFMA/Ru-Cat (10 mL, 0.5 mg/mL) slowly and performed dialysis against H_2_O for 5 days to prepare nanoreactors of PEG-*b*-PBMA/Ru-Cat and PEG-*b*-PPFMA/Ru-Cat. Transmission electron microscopy (TEM) images confirmed that both PEG-*b*-PBMA/Ru-Cat and PEG-*b*-PPFMA/Ru-Cat formed vesicles. The membrane thickness of vesicles was found to be 28 nm and 27 nm for the PEG-*b*-PBMA/Ru-Cat and PEG-*b*-PPFMA/Ru-Cat vesicles, respectively. Dynamic light scattering (DLS) analysis suggested that the hydrodynamic diameters (*D*h) of vesicles PEG-*b*-PBMA/Ru-Cat and PEG-*b*-PPFMA/Ru-Cat were 281 nm and 295 nm, respectively. They studied the oxidation of 4-methoxyphenylboronic acid to 4-methoxyphenol (Figure 10A) in a vesicle system under irradiation of blue lights. The yield of 4-methoxyphenol reached approximately 93% in 20 h when the reaction was performed in the vesicles of PEG-*b*-PPFMA/Ru-Cat (Table 3). In contrast, the yield for the reaction was found to be 52%, even after 30 h, when the reaction occurred in the vesicles of PEG-*b*-PBMA/Ru-Cat (Table 3).

Zou et al. (2012) reported a similar reaction using 2 mol% Ru(bpy)_3_ as a catalyst in DMF. The yield was 94% after 28 h. However, in aqueous media, the yield was reduced to 60% even after 96 h, which was much lower than that found in vesicles of PEG-*b*-PPFMA/Ru-Cat. To prove the general role of fluorinated vesicles in accelerating the oxidation reaction, Que et al. (2020) used different substrates with structure modulation. The yields were found to be approximately 90% and 92% for 4-propylphenylboronic acid and 4-hexylphenylboronic acid, respectively, which were much higher than those found in non-fluorinated vesicles (33% and 42%) (Table 3). These results confirmed that the fluorinated vesicular nanoreactors embedded with Ru-Cat could act as an excellent host for oxidation reactions in aqueous media. The properties of solvents or local surroundings where a photooxidation reaction occurs may be changed in vesicles. The viscosities and dielectric constants of solvents can influence the decay process of produced singlet oxygen and the rate of oxygen diffusion (Young et al., 1971). As fluorinated and non-fluorinated vesicles had similar shape and size, it can be said that the fluorinated membrane of vesicles embedded with photocatalyst had a higher local concentration of oxygen and rate of diffusion compared to the non-fluorinated membrane due to the special fluorine effect (Que et al., 2016). The main advantage of the fluorinated nanoreactors was that they could be recycled and reused for at least five cycles with a slight decrease in efficiency. In a recent work, Higashida et al. (2023) developed a conjugate system in which the hydrophobic Ru(II) dye-sensitized Pt-TiO_2_ nanoparticle photocatalyst is impregnated into 1,2-dipalmitoyl-*sn*-glycero-3-phosphocholine (DPPC) lipid bilayer vesicle membranes. The photocatalytic activity of H_2_ production in this vesicle was found to be three times faster than that in the absence of DPPC vesicles alone. The ability of DPPC vesicles to disperse the hydrophobic catalyst is the key factor in obtaining increased activity of photocatalytic H_2_ production in aqueous solution.

### 2.4 Metallo catalytic reduction of 5-hydroxymethylfurfural

2,5-Dimethylfuran is a potential biomass fuel due to its appropriate energy density, boiling point, octane value, hydrophobic property, and production efficiency (Hoang et al., 2021; Qian et al., 2015). 2,5-DMF can be synthesized from 5-hydroxymethylfurfural (HMF) obtained from biomass (Chen et al., 2020). Pd/C, Ru/C, Ru/Co_3_O_4_, and others have been reported to catalyze the reduction reaction of HMF to 2,5-DMF (Chen et al., 2020; Thananatthanachon and Rauchfuss, 2010; Yang et al., 2017; Zhang et al., 2012).

In this context, Shimanouchi et al. (2022) studied the contribution of vesicles in the reduction of HMF to 2,5-DMF (Figure 10B). Conventional metal catalysts combined with vesicles are expected to improve the solvent environment for the reduction of HMF. They used four different phospholipids, including 2-dipalmitoyl-*sn*-glycero-3-phosphocholine (DPPC), 1,2-dimyristoly-*sn*-glycero-3-phosphocholine (DMPC), 1-palmitoyl-2-oleoyl-*sn*-glycero-3-phosphocholine (POPC), and 1,2-dioleoyl-*sn*-glycero-3-phosphocholine (DOPC) (Figure 2). They also used non-phospholipid vesicles composed of Span and Tween series. The size of the vesicles was adjusted to be 100 nm. Calcein leakage experiments and cryo-TEM indicated that many vesicles were ruptured, leaving part of the vesicles intact, which kept their structure. HMF (2.0 × 10^−2^ mol) was dissolved in 10 mL of THF, and then Pd/C (metal: 4.7 × 10^−5^ mol) was added to the solution. The reaction mixture was incubated for 6–24 h at 60 °C, which is a mild reaction condition compared to the conventional 130 °C. The conversion of HMF to 2,5-DMF was monitored by high-performance liquid chromatography. The effect of vesicles on the conversion was observed by adding 0.5 mL of vesicle suspension (lipids 2 × 10^−6^ mol) to 10 mL of HMF in THF solution (water content 4.7 vol%). Vesicle-combined metal-supported catalysts (VCMSCs) were characterized with the negative staining method. Some vesicles were found to be at the surface of Pd/C. The effect of vesicles on the electrochemical properties of Pd/C was determined by the cyclic voltammogram method. It was found that the electrochemical property of Pd/C remained unchanged after combination with vesicles. The yield using VCMSCs was found to be similar to that in Pd/C alone. The yield of 2,5-DMF was regulated by the lipid composition, whereas the conversion of HMF was not dependent on composition that much. The order of the 2,5-DMF production was DPPC ∼ DMPC < DOPC < POPC. The order of yield can be related to the membrane fluidity or hydration property of vesicles. The phospholipid vesicles have much more polarized bound water than the Span/Tween vesicles (Shimanouchi et al., 2011). The relationship between lipid composition in VCMSCs and the reactivity suggested that the polarized bound water contributed to the reaction process of HMF to 2,5-DMF.

A recent work has shown that the conversion of 5-hydroxymethylfurfural to 2,5-furandicarboxylic acid (FDCA) (Figure 10C) can be catalyzed by using a catalyst of PtBi-activated carbon (PtBi/C) combined with vesicles made of phospholipids under mild temperature and non-basic conditions (Shimanouchi et al., 2025). The highest yield of FDCA (54.4% ± 2.7%) was obtained in DOPC vesicles. The yield of FDCA was found to be dependent on the hydration nature of vesicles. FDCA is an alternative compound for terephthalic acid in the production of poly (ethylene terephthalate). The conventional method for the conversion of HMF to FDCA requires a basic condition, such as sodium hydroxide. In a vesicle-mediated reaction, the bound water acts as an oxygen source, giving an alternative to the base. Zhao et al. (2021) developed a copper nanoparticle encapsulated in a vesicle-like boron-doped carbon nitride (BCN) matrix (BCN@Cu), where electrochemical reduction of nitrate (E-NIRR) to ammonia has shown excellent catalytic activity.

### 2.5 Decarboxylation reaction

**FIGURE 11:**
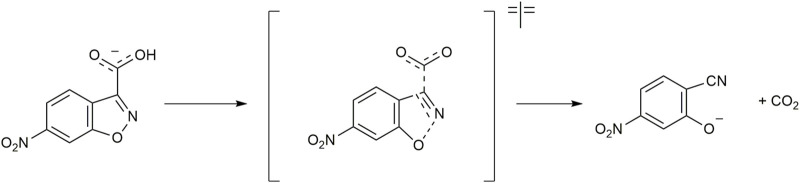
Decarboxylation reaction of 6-nitrobenzisoxazole-3-carboxylate (6-NBIC).

Decarboxylation reactions have gained interest as they are involved in biological transformations like the biosynthesis of alcohols, terminal olefins, and nucleobases. However, without any activating agent, these reactions are slow (Zhou et al., 2021). The decarboxylation reaction of the 6-nitrobenzisoxazole-3-carboxylate anion (6-NBIC) (Figure 11) is a unimolecular reaction that is very sensitive to changes in the local polarity at the binding sites of the reactant (Grate et al., 1993).

When the reaction is performed in apolar solvents, the rate constant for the decarboxylation reaction is reduced due to the formation of an ion pair, whereas an increase in polarity would increase the rate constant. However, hydrogen bonding may contribute to a decrease in the rate constant through the stabilization of the initial state. Cationic ammonium vesicles were found to be an excellent medium for decarboxylation reaction by accelerating the reaction 1000 times relative to that in pure water (Kunitake et al., 1980). It has been shown that catanionic vesicles (a mixture of dodecyltrimethylammonium bromide (DTAB) and sodium dodecyl sulfate (SDS)) can further accelerate the decarboxylation of the 6-NBIC reaction by nearly 30% compared to cationic micelles composed of CTAB (Talhout and Engberts, 1997). The rate of the decarboxylation reaction may be influenced by the binding of the carboxylate onto the bilayer and the chemical transformation (decarboxylation) of the carboxylate-bilayer ion pair. The appropriate microenvironment, such as the electrostatic potential of the bilayer membrane surface, acts as the perfect host for the reaction to take place. In addition to that, the interaction between the cationic head group and the 6-NBIC anion helps in binding at the vesicular interface. It reduces the chances of hydrogen bonding formation between water and the substrate, which also increases the rate constant. To understand the effect of additives on decarboxylation in vesicles, Jongejan et al. (2006) performed the decarboxylation of 6-NBIC in di-*n*-hexadecyldimethylammonium bromide (DHAB) vesicles in the presence of different additives, such as the monohydric alcohols *n*-dodecanol (DD) and *n*-hexadecanol (HD), as well as two glycerol ethers (with one or two alkyl tails) and cholesterol. They also used C12-tailed surfactants with sugar headgroups and sugars without a hydrophobic anchor, such as glucose (Glu), maltose (Mal), and trehalose (Treh), as an additive. Most of the additives had inhibited the decarboxylation reaction of 6-NBIC compared to the reaction in vesicles without any additives. The inhibition was greatest with cholesterol. In contrast, *n*-dodecyl-β-maltoside and trehalose accelerated the decarboxylation. This increase in the rate constant may be due to the replacement of water from the interface by the sugar hydroxyl groups. The hydroxyl groups of the glycerol ether may also be able to displace water, but due to the lesser number of hydroxyl groups at the interface, the positive effect is less pronounced.

### 2.6 Diels–Alder reaction

**FIGURE 12:**
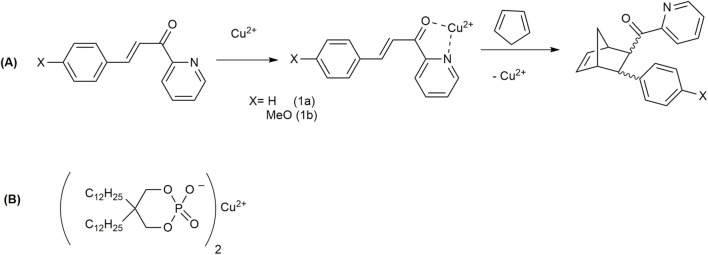
**(A)** Reaction of cyclopentadiene with 3-phenyl-1-(2-pyridyl)-2-propen-1-one (1a) and 3-(4-methoxyphenyl)-1-(2-pyridyl)-2-propen-1-one (1b). **(B)** Structure of Cu(dDP)_2_.

The Diels–Alder cycloaddition reaction is one of the most important organic reactions, as it can construct six-membered carbocyclic and heterocyclic systems with high regio- and stereoselectivity (Soares et al., 2022). This click reaction occurs under mild conditions (Yeingst et al., 2024). Rispens and Engberts (2001) performed Diels–Alder reactions of azachalcone derivatives with cyclopentadiene (2) (Figure 12A) using vesicular catalysis.

The vesicle host used was Cu(dDP)_2_ (Figure 12B), prepared from a sodium salt of 5,5-di-*n*-dodecyl-2-hydroxy-1,3,2-dioxaphosphorinan-2-one (NadDP) and cupric chloride. Most of the vesicles were 40 nm, although a very few larger (200 nm) ones have been found. They developed a combination of Lewis acid catalysis and vesicular catalysis. The efficiency of the metallo-vesicular catalysis is extremely high even at a very low concentration of Cu(dDP)_2_(0.1 mM), involving a rate increase relative to the uncatalyzed reaction in acetonitrile of a factor of 1 × 10^6^. Although the maximum rate of reaction was found to be 1.5–2 times higher in Cu(DS)_2_ (DS is di-*n*-dodecyl sulfate) micelles than that in Cu(dDP)_2_ vesicles, the catalytic effect was found at much lower concentrations with a factor of 10–20 in the metallo-vesicles than that in the metallo-micelles. This fact has made the vesicle reactor a greener host for reactions due to the lower critical vesicle concentration (cvc) of Cu(dDP)_2_ compared to the critical micellar concentration (cmc) of Cu(DS)_2_. Hydrophobic microdomains can be formed at lower concentrations of Cu(dDP)_2_, where the reactants can bind. Cyclopentadiene binds into the hydrophobic core of the vesicle bilayer, and the dipolar dienophile binds at the surface of the bilayer by forming a complex with copper ions. The relatively lower rate of reaction in the vesicle than that in micelles may arise from the greater hydrophobicity of the inner core of the vesicles, resulting in more shielding of cyclopentadiene from the dienophile. The smaller vesicle with more curvature allows better interaction of the hydrophobic diene with the Cu(II)-complexed dienophile, making it a better catalytic host than the larger one.

### 2.7 Kemp elimination

The Kemp elimination is a well-known reaction in which a catalytic base abstracts a proton from the benzo[*d*]isoxazole ring, resulting in ring-opening to form the cyanophenol product. The Kemp reaction is applicable for probing biochemically relevant reaction conditions (Chen et al., 2018). In this reaction, proton removal by the base and N-O bond breakage occur through a concerted mechanism.

**FIGURE 13:**
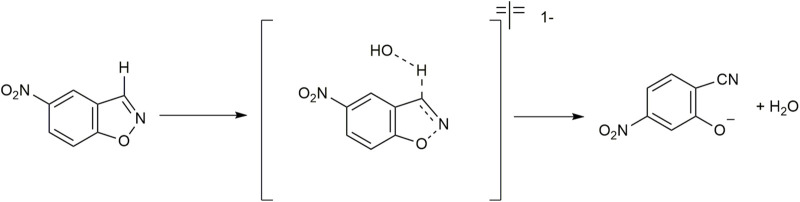
Kemp elimination reaction.

Klijn and Engberts (2003) monitored the deprotonation of 5-nitrobenzisoxazole (Figure 13) by hydroxide in vesicles formed with dimethyldioctadecylammonium chloride (C_18_C_18_^+^) and catanionic vesicles formed by C_18_C_18_^+^ and sodium didecyl phosphate (C_10_C_10_^−^) to a concentration of 16 mM. The C_18_C_18_^+^ vesicles were found to be “lens-shaped.” With an increase in concentration of C_10_C_10_^−^, the curvature of vesicles increased. At 70 mol% C_10_C_10_^−^, only spherical vesicles are observed. The reaction is efficiently catalyzed in C_18_C_18_^+^ vesicles. The difference in chain length was chosen to avoid precipitation of the catanionic mixture. No catalysis was observed in anionic vesicles. The detailed kinetic analysis has shown that the rate constant of the reaction in the C_18_C_18_^+^ vesicle is nearly 65 times larger than that in water. However, the addition of sodium dodecyl phosphate (C_10_C_10_^−^) resulted in a reduction of observed catalysis. With an increase in the total concentration of the amphiphile, there was initially a sharp increase in k_obs_. After reaching a maximum value depending on the percentage of C_10_C_10_^−^ in the bilayer, k_obs_ decreased slowly. This observation is a typical characteristic of micellar and vesicular catalysis of bimolecular reactions. The experiments with Reichardt’s ET probe and pyrene fitting model indicated that the counterion (OH^−^) binding to the cationic C_18_C_18_^+^ amphiphiles is reduced with the addition of C_10_C_10_^−^ as local positive charge density was reduced. Catalytic efficiency was also reduced with the addition of C_10_C_10_^−^. In a related work, Klijn and Engberts (2004) also observed the effect of additives like mono-, di-, and poly-hydric alcohols (pyranosides) on vesicular catalysis. Pérez-Juste et al. (2000) showed that the catalytic efficiencies of the vesicles at 25 °C follow the order: DDAB (didodecyldimethylammonium bromide) > DODAC (dioctadecyldimethylammonium chloride) > DODAB (dioctadecyldimethylammonium bromide) (k_max_/k_w_; 860:550:160, respectively). They found that the rate of catalytic reaction involving less strongly hydrated hydroxide ions can result in overall rate enhancement up to a factor of 850. The relatively low efficiencies of bilayers with two C_18_ chains may be due to membrane rigidity. The phase transition temperatures of the above-mentioned bilayers indicated the rigid state of DODAB and DODAC bilayers, whereas the DDAB bilayer was in the fluid (liquid crystalline) state. In the case of DODAB and DODAC vesicles, binding cholesterol to the bilayer further increases the catalytic efficiency.

### 2.8 Multicomponent reactions

Multicomponent reactions (MCRs) are powerful one-pot synthetic tools to produce complex organic molecules in a single step. Isocyanide-based multicomponent reactions like Passerini and Ugi reactions are convenient for the synthesis of peptidomimetics (Paprocki et al., 2018).

**FIGURE 14:**
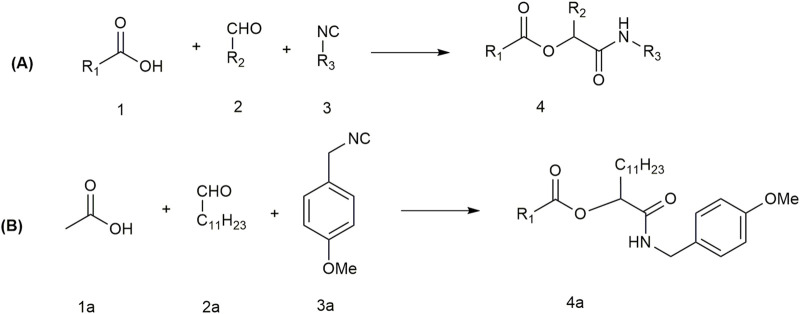
**(A)** Passerini reaction of carboxylic acid (1), aldehyde (2), and an isocyanide (3) to form an *α*-acyloxy carboxamide. **(B)** Passerini reaction of acetic acid (1a), dodecyl aldehyde (2a), and *p*-methoxybenzyl isocyanide (3a).

The Passerini reaction is a multicomponent reaction where α-acyloxyamides are produced from the reaction of an isocyanide, an aldehyde or ketone, and a carboxylic acid (Figure 14A). α-Acyloxy carboxamides are an important natural product due to their biological and pharmacological activities (Wu et al., 2023). The Passerini reaction is applied in fields like natural product chemistry, biomedical science, pharmaceuticals, polymer science, and many other fields. The reaction is conventionally done in aprotic organic solvents such as dichloromethane or toluene, but they are toxic and carcinogenic. To overcome this problem, Pirrung and Sarma (2004) performed the reaction in green solvents, such as water.

Later, Paprocki et al. (2015) observed that for hydrophobic starting materials, the reaction yield was increased in the presence of vesicles prepared from dioctadecyldimethylammonium bromide (DODAB) compared to the yield without DODAB or in dichloromethane. First, they performed the multicomponent reaction using acetic acid (1a), dodecyl aldehyde (2a), and *p*-methoxybenzyl isocyanide (3a) (Figure 14B). Among different organic solvents, dichloromethane has proved to be the best solvent for the reaction. In DCM, the yield was 59%, whereas in green solvent water or in aqueous phosphate buffer saline (PBS), the yields were 32% and 33%, respectively (Table 3). To avoid the use of toxic organic solvents, they aimed to improve the reaction efficiency in aqueous solvents. The low solubility of organic substances in water was the main disadvantage. They added micelle-forming surfactants like sodium dodecyl sulfate (SDS), sodium bis(2-ethylhexyl) sulfosuccinate (AOT), and vesicle-forming surfactants didodecyldimethylammonium bromide (DDAB) and dioctadecyldimethylammonium bromide (DODAB) to observe the reaction efficiency in a self-aggregated system. The surfactants were added at 20 mol% of the individual starting materials. For comparison, the effect of a few other additives was also tested. Anionic surfactants reduced the reaction efficiency, which may be due to repulsive interactions between the acetate ions (deprotonated form of 1a) and the negatively charged surface of the micellar or vesicular aggregates. In contrast, in the presence of non-ionic surfactants (Span 60, Tween 80, and Triton X-100), the reaction yields were between 43% and 47%, which was higher than in PBS (33%). The reaction yields were even increased up to 48%–58% in the presence of cationic surfactants, particularly DODAB (Table 3). This enhancement may be due to two major effects: (i) increased solubility of the reactants in the hydrophobic part of the bilayered vesicular aggregates and (ii) electrostatic attractions between the cationic surface of the vesicles and the acetate ions, resulting in local concentrations of the reacting molecules in the region of the vesicle bilayers. This accelerating effect is due to the incorporation of cationic DODAB, as the addition of NaCl or NaBr could not increase the reaction yield. They performed 16 different reactions using different isocyanides, carboxylic acids, and different aldehydes in DODAB vesicles and DCM. In every instance, the reaction was as efficient as that in DCM or, in some cases, even better. Therefore, vesicles can be used as a green alternative to organic solvents for multicomponent reactions. Madej et al. (2017) performed a 4-component Ugi reaction of phenylacetic acid, isovaleraldehyde, *p*-methoxybenzylamine, and *p*-methoxybenzyl isocyanide in different solvent systems. Compared to commonly used organic solvents, surfactant aggregates, especially DDAB vesicles, were found to be a better solvent for the Ugi reaction, where the yield is even higher than that of the best conventional solvent, methanol (Table 3). Synthetically important multicomponent reactions can be performed in aqueous medium with improved yield using vesicular aggregates.

## 3 Enzymatic reactions in vesicles

Enzymes are natural catalysts for several organic reactions, showing excellent levels of efficiency and selectivity in an aqueous environment. Their catalytic activity is modulated by physico-chemical stimuli, such as small molecules, light, temperature, and pH (Motlagh et al., 2014). Enzymes are very sensitive to minute environmental changes, so it is difficult to retain both the structure and activity of enzymes. These limitations can be overcome by encapsulation of enzymes in cell-like liposomal carriers (Minteer, 2017; Mohammadi et al., 2021). The presence of charges in the vesicle membrane results in an adsorption of the charged enzyme onto the interior or exterior site of the vesicle bilayers (Walde and Ichikawa, 2001). The encapsulated enzyme must be released from the vesicles before the reaction so that it can catalyze a reaction occurring outside of the vesicles. On the other hand, the entrapped enzyme molecules may catalyze reactions inside the vesicles without any release. In the second case, the substrate must enter through the vesicle bilayers to meet the enzyme within the vesicles. Enzyme-containing lipid vesicles have a wide range of applications in the medical or biomedical fields (Berumen Sánchez et al., 2021; Girase et al., 2025; Hasegawa et al., 2022; Ma et al., 2023; Walde and Ichikawa, 2001) and in the food industries (Emami et al., 2016).

In this regard, Li et al. (2007) investigated the entrapment of amyloglucosidase (AMG) from *Aspergillus niger* into multilamellar vesicles (MLVs) and large unilamellar vesicles (LUVs) made of dipalmitoylphosphatidylcholine (DPPC) (Figure 2). Vesicles incorporating AMG were isolated from the free enzyme solution by centrifugation. The kinetics of soluble starch hydrolysis by free AMG in aqueous solution, as well as entrapped enzyme within vesicles in aqueous suspension, were measured using a Michaelis–Menten kinetic model with product inhibition. The rate of hydrolysis by entrapped AMG was found to be relatively low compared to that by free AMG. The reason could be the low permeability of the substrate across the vesicle bilayer or because of the low enzyme activity inside the liposomes. However, when the enzyme solution was incubated at the hydrolysis temperature in sealed tubes, the AMG entrapped inside MLV and giant unilamellar vesicles (GUVs) retained their activity for a much longer period than the free enzyme in aqueous media. The authors were also able to recycle the entrapped enzyme with retention of 60% activity after three cycles. Therefore, it can be said that entrapped enzymes were much more stable than free enzymes.

Blocher et al. (1999) investigated the kinetic behavior of the α-chymotrypsin-catalyzed hydrolysis of the two *p*-nitroanilide substrates, succinyl-L-Ala-L-Ala-L-Pro-L-Phe-*p*-nitroanilide (Suc-Ala-Ala-Pro-Phe-pNA) and benzoyl-L-Tyr-p-nitroanilide (Bz-Tyr-pNA), in an aqueous solution of α-chymotrypsin and 1-palmitoyl-2-oleoyl-sn-glycero-3-phosphocholine (POPC) vesicles containing entrapped α-chymotrypsin. For the vesicle system, the substrate was added to the bulk, exovesicular aqueous phase. Bz-Tyr-pNA can penetrate across the bilayer shell of the vesicle and reach the vesicle’s aqueous interior, where α-chymotrypsin catalyzes a hydrolysis reaction. In contrast, Suc-Ala-Ala-Pro-Phe-pNA cannot permeate the phospholipid bilayer. The compartmentalization and permeability properties of the vesicle determine the substrate selectivity. The substrate permeability coefficient for Bz-Tyr-pNA by parametric fitting was found to be 2.45 × 10^−7^ cm/s.

Kato et al. (2003) studied the enzymatic activity and stability of D-fructose dehydrogenase (FDH) and sarcosine dehydrogenase (SDH) immobilized onto giant vesicles made up of the surfactants Span 80 and Tween 80 and the phospholipid lecithin (phosphatidylcholine from soybeans).

The SDH activity was measured using sarcosine substrate in the potassium phosphate buffer (pH 7.5) with the help of (oxidized) PMS (1-methoxy-5-methylphenazinium methylsulfate) and nitroblue tetrazolium chloride (NBT), leading to the formation of glycine, formaldehyde, and the “NBT-formazan.”

The activity of FDH was measured by the conversion of D-fructose into 5-dehydro-D-fructose in the McIlvaine buffer (pH 4.5) in the presence of 10 mM K_3_[Fe(CN)_6_]. For comparison, the catalytic activity and enzymatic stability were also measured for enzymes in a vesicle-free solution. The enzyme activity and stability of FDH and SDH enzymes were found to be considerably increased upon immobilization of the enzymes on vesicles. However, immobilization of sorbitol dehydrogenase from *Gluconobacter suboxydans* (SODH) did not improve activity or stability, unlike FDH and SDH.

Zhang et al. (2017) investigated the activity of horseradish peroxidase (HRP) using *p*-phenylenediamine (PPD) as electron donor substrate and hydrogen peroxide (H_2_O_2_) as oxidant in aqueous solution, as well as inside ≈180 nm-sized lipid vesicles prepared from POPC (1-palmitoyl-2-oleoyl-sn-glycero-3-phosphocholine).

PPD (0.05–1.50 mM) was converted to Bandrowski’s base using 0.5 nM HRP, 0.2 mM H_2_O_2_ in a pH 7 buffer. HRP-catalyzed oxidation of PPD occurred inside the vesicles, as PPD has high POPC bilayer permeability. It was also observed that HRP molecules in vesicles are stable for at least 1 month without any considerable HRP leakage, if stored at 4 °C. In contrast, when the bilayer-impermeable substrate ABTS^2−^(2,2′-azino-bis(3-ethylbenzothiazoline-6-sulfonate)) was used, the oxidation of ABTS^2−^ inside the vesicles did not occur. In another work, they used mixed anionic vesicles (diameter ≈80 nm) constituted of sodium dodecylbenzenesulfonate (SDBS) and decanoic acid (1:1, molar ratio) as a template for polymerization of aniline to yield the conductive emeraldine salt form of polyaniline using horseradish peroxidase/hydrogen peroxide (H_2_O_2_) (Guo et al., 2009). The oxidative polymerization of aniline can produce different polymeric products, the morphology and physico-chemical properties of which depend on several factors, such as the type of oxidant used, the concentrations of aniline, temperature, and acidity (Sapurina and Stejskal, 2008). The template plays a crucial role in regulating the morphology of polyaniline (PANI). Major templates are negatively charged polymers, micelles, or vesicles. In the absence of vesicles under identical reaction conditions, the conductive form of PANI was not formed. However, an SDBS/decanoic acid (1:1) vesicle cannot be used for the enzymatic polymerization of aniline at low temperatures due to the precipitation of vesicles. To overcome this problem, they used AOT (bis-(2-ethylhexyl) sulfosuccinate) vesicles as a host for the horseradish peroxidase/H_2_O_2_-triggered polymerization of aniline to yield the green emeraldine salt form of polyaniline (Guo et al., 2011).

Lefrançois et al. (2020) investigated bi-enzymatic reactions involving a glucose oxidase (GOx) and the horseradish peroxidase (HRP) in giant unilamellar vesicles (GUVs) formed with soybean polar extract, a natural mix of phospholipids like 45.7 wt/wt% phosphatidylcholine, 22.1 wt/wt% phosphatidylethanolamine, 18.4 wt/wt% phosphatidylinositol, and 6.9 wt/wt% phosphatidic acid. They used two substrates, glucose and Amplex Red. The GUVs are semi-permeable compared to other purely synthetic GUVs, which allows the small molecules, that is, the enzymatic substrates, to diffuse from the outer solution toward the internal volume and activate the enzymatic reactions.

Ridgway-Brown et al. (2025) encapsulated urease within DOPC lipid vesicles with a 164 ± 3 nm diameter. Urease catalyzes the hydrolysis of urea to produce ammonia and carbon dioxide. They observed that a thinner membrane prolonged reaction times. This may be due to reduced membrane permeability of protons with thicker membranes, which increases the localized base-catalyzed feedback mechanism inside the vesicles. They also showed how proton movement significantly impacts internal reactions by changing bilayer thickness, adding ion transporters, and varying buffers.

Papain is a useful enzyme that helps digest proteins. Takahashi et al. (2024) developed liposomes composed of DOPC and *N*-(aminopropylpolyoxyethylene oxycarbonyl)-1,2-distearoyl-*sn*-glycero-3-phosphoethanolamine (DSPE-PEG-NH_2_) as a host for papain. Papain was covalently attached through tris-succinimidyl aminotriacetate (TSAT) to liposomes with a 5 mol% poly (ethylene glycol)-tethered lipid with a reactive amino group. The papain-conjugated liposome catalyzed the hydrolysis of *N*_α_-benzoyl-l-arginine-4-nitroanilide hydrochloride (BAPNA) at pH = 5.0–7.0. The activity of liposome–papain increased with increasing temperature from 25 °C to 50 °C. Slight conformational changes in both its secondary and tertiary structures of conjugated papain were found from circular dichroism and intrinsic fluorescence measurements. This liposome–PEG–papain conjugate had also shown the digestion activity of casein at 37 °C and pH = 7.6 (Takahashi et al., 2025).

Laccase is an important enzyme that can remove organic contaminants, but its stability and effectiveness are reduced by photo-induced degradation processes. To solve this problem, Luna et al. (2025) immobilized laccase in unilamellar and multilamellar vesicles of dioleoyl phosphatidylcholine (DOPC), and enzyme activity was investigated spectroscopically for both free and entrapped laccase in vesicles before and after UVB irradiation.

They used syringaldazine as a substrate for laccase activity studies, as it can permeate through the bilayer. Oxidation of syringaldazine to tetra-methoxy-azo-bis-methylene quinoline was catalyzed by laccase. Laccase entrapped in DOPC vesicles was found to retain its activity compared to free enzyme. This simple process is suitable for bioremediation on a large scale. Martí et al. (2012) observed that laccases embedded in phosphatidylcholine liposomes showed much more retention of activity than that of free native enzymes. Oxidation of 2,2՛-azino-bis(3-ethylbenzthiazoline-6-sulfonate) (ABTS) was studied to monitor enzyme activity. After 3 days of heating, the loss of activity of laccases was found to be less than 10% in vesicles compared to 40%–60% of loss for free-laccases.

Glucose oxidase (GO) catalyzes the oxidation of glucose, producing gluconic acid and hydrogen peroxide. Wang et al. (2003) prepared glucose oxidase-containing liposomes (GOL) composed of phosphatidylcholine (PC), dimyristoyl L-α-phosphatidylethanolamine (DMPE), and cholesterol (Chol) by the extrusion method and then covalently immobilized them in the glutaraldehyde-activated chitosan gel beads.

GO catalyzes the oxidation of glucose to gluconic acid and hydrogen peroxide. This hydrogen peroxide was quantified by the HRP-catalyzed formation of oxidized *o*-dianisidine to measure the GO activity. Immobilized GOL gel beads (IGOL) were prepared with optimal immobilization conditions when highest possible activity efficiency of the IGOL, and the lowest possible leakage of the entrapped GO were found to be at liposome composition: PC/DMPE/Chol = 65/5/30 (molar percentage); liposome size: 100 nm; glutaraldehyde concentration: 2% (w/v); immobilizing temperature: 4 °C; and immobilizing time: 10 h. They observed that the stability of the GO molecules embedded in the IGOL and GOL is considerably higher than that in the immobilized GO without liposome (IGO) and free GO. For recycling, IGOL is preferable to the GOL. The activity of GO was further improved here by incorporating the channel protein OmpF from *Escherichia coli* into the liposome membrane, which increased the permeability of the liposome membrane toward glucose substrate. They further improved the enzyme activity using catalase (CA) from *A. niger*, which decomposes the hydrogen peroxide produced, as hydrogen peroxide inhibits the activity of GO (Yoshimoto et al., 2005). They found a notable protective effect of the bilayer membrane on CA inside the liposomes at 40 °C. After 72 h, the CA activity was higher than 60% for liposomes containing GO and CA (GOCAL), while it was less than 20% for free CA. Furthermore, when OmpF was incorporated into GOCAL membranes (GOCAL-OmpF), the activity of GO inside GOCAL-OmpF increased up to 17 times in comparison with that inside GOCAL due to an increased permeability of glucose permeation across the liposome bilayer, without any leakage of GO or CA from the liposomes.

Nagata et al. (2023) covalently attached bovine carbonic anhydrase (BCA) enzyme molecules to liposomes. The liposomes comprised DOPC, *N*-(methylpolyoxyethylene oxycarbonyl)-1,2-distearoyl-*sn*-glycero-3-phosphoethanolamine (DSPE-PEG), and DSPE-PEG-NH_2_. They used 4-nitrophenyl acetate (p-NA) as a substrate. BCA–liposome conjugate was found to have similar catalytic activity and storage stability as free BCA. At 5 °C, liposome–BAH–BCA can catalyze the hydration of carbon dioxide to hydrogen carbonate.

## 4 Conclusion

Several research works have already proved that vesicles can act as nanoscale reaction vessels to obtain organic reactions in water. Although in some instances they can also inhibit organic reactions, such an outcome is not disappointing if a proper explanation is obtained. Vesicles have been proven to be an excellent host for enzymes by providing improved enzyme stability and controlled reaction conditions. More studies are needed to engineer vesicular aggregates to catalyze organic reactions in water, as well as to perform complex cellular reactions as an artificial cell. In this way, supramolecular chemistry could be a tool to achieve green chemistry.
